# Diagnostic value of immune indicators and cytokines in sepsis after percutaneous nephrolithotomy (PCNL)

**DOI:** 10.5937/jomb0-60616

**Published:** 2026-04-15

**Authors:** Yiheng Jin, Yamei Li, Xiaoyu Zhang, Bing Han, Xingshi Yan

**Affiliations:** 1 Wuhan University, Tongren Hospital of Wuhan University, Department of Emergency, Wuhan City, China; 2 The 940th Hospital of Joint Logistics Support Force of Chinese People's Liberation Army, Department of Emergency, Lanzhou City, China; 3 Peking University Shenzhen Hospital, Laboratory Department, Shenzhen City, China; 4 Air Force Medical University, Air Force 986 Hospital, Department of Emergency, Xi'an City, China

**Keywords:** sepsis, percutaneous nephrolithotomy, cytokine, immune indicators, sepsa, perkutana nefrolitotomija, citokini, imunološki parametri

## Abstract

**Background:**

To analyse the changes in and diagnostic value of immune indicators and cytokines in patients with sepsis after percutaneous nephrolithotomy (PCNL).

**Methods:**

Clinical information was gathered from 405 patients with calculi who underwent PCNL at our facility between January 2021 and December 2024. The patients were divided into a sepsis group (12 patients) and a non-sepsis group (393 patients) based on whether sepsis occurred after the operation. The levels of CD4+/CD 8 + cells, the neutrophil-to-lymphocyte ratio (NLR), soluble trigger receptor-1 (sTREM-1) in myeloid cells, procalcitonin (PCT), tumour necrosis factor-alpha (TNF-<span style="color: rgb(32, 33, 34); font-family: sans-serif; font-size: 16px; background-color: rgb(248, 249, 250);">α</span>), and interleukin-6 (IL-6) were measured in the two patient groups. The predictive value of the above indicators for sepsis was analysed via receiver operating characteristic (ROC) curves.

**Results:**

The number of patients with staghorn calculi in the sepsis group was greater than that in the nonsepsis group (P&lt; 0.05). The NLR and sTREM-1 levels were greater in the sepsis group than in the nonsepsis group (all P&lt; 0.05), and the CD 4+/CD 8+ ratio was lower in the sepsis group than in the nonsepsis group (P&lt; 0.05). The levels of serum IL-6, TNF-<span style="color: rgb(32, 33, 34); font-family: sans-serif; font-size: 16px; background-color: rgb(248, 249, 250);">α</span> and PCT in the sepsis group were greater than those in the nonsepsis group (all P&lt; 0.05). The area under the curve (AUC) for the combined prediction of sepsis after PCNL by each index was 0.996, with a predictive sensitivity of 91.3% and a specificity of 98.4%.

**Conclusions:**

The NLR, sTREM-1, IL-6, TNF-<span style="color: rgb(32, 33, 34); font-family: sans-serif; font-size: 16px; background-color: rgb(248, 249, 250);">α</span> and PCT in patients with sepsis after PCNL increased, whereas the CD 4+/CD 8+ ratio decreased. The combined detection of these levels is beneficial for guiding the early clinical prediction of postoperative sepsis in patients undergoing PCNL.

## Abstract (sr)

**Uvod:**

Cilj je bio da se analiziraju promene i dijagnostički značaj imunoloskih parametara i citokina kod pacijenata sa sepsom nakon perkutane nefrolitotomije (PCNL).

**Metode:**

Klinički podaci prikupljeni su od 405 pacijenata sa kalkulusima, koji su u nasoj ustanovi bili podvrgnuti PCNL-u u periodu od januara 2021. do decembra 2024. Pacijenti su podeljeni u grupu sa sepsom (12 pacijenata) i grupu bez sepse (393 pacijenta), u zavisnosti od pojave sepse nakon operacije. U obe grupe određivani su nivoi CD4+/CD 8 + celija, odnos neutrofila i limfocita (NLR), rastvorljivog receptora okidača-1 (sTREM-1) u mijeloidnim celijama, prokalcitonina (PCT), faktora nekroze tumora alfa (TNF-<span style="color: rgb(32, 33, 34); font-family: sans-serif; font-size: 16px; background-color: rgb(248, 249, 250);">α</span>) i interleukina-6 (IL-6). Prediktivna vrednost ovih parametara za pojavu sepse analizirana je pomocu ROC (receiver operating characteristic) krivih.

**Rezultati:**

Broj pacijenata sa koralnim kalkulusima bio je veći u grupi sa sepsom nego u grupi bez sepse (P&lt; 0,05). Vrednosti NLR-a i sTREM-1 bile su više u grupi sa sepsom u poređenju sa grupom bez sepse (sve P&lt; 0,05), dok je odnos CD 4+/CD 8+ bio niži u grupi sa sepsom (P&lt; 0,05). Nivoi serumskog IL-6, TNF-<span style="color: rgb(32, 33, 34); font-family: sans-serif; font-size: 16px; background-color: rgb(248, 249, 250);">α</span> i PCT takođe su bili viši u grupi sa sepsom (svi P&lt; 0,05). Površina ispod krive (AUC) za kombinovanu predikciju sepse nakon PCNL-a iznosila je 0,996, sa senzitivnošću od 91,3% i specifičnošću od 98,4%.

**Zaključak:**

Kod pacijenata sa sepsom nakon PCNL-a zabeleženo je povećanje vrednosti NLR-a, sTREM-1, IL-6, TNF-<span style="color: rgb(32, 33, 34); font-family: sans-serif; font-size: 16px; background-color: rgb(248, 249, 250);">α</span> i PCT, uz smanjenje odnosa CD4+/CD 8 + . Kombinovano određivanje ovih parametara može biti od značaja za ranu kliničku predikciju postoperativne sepse kod pacijenata podvrgnutih PCNL-u.

## Introduction

The main treatment for upper ureteral calculi and kidney stones is percutaneous nephrolithotomy (PCNL), which causes less trauma to the human body and enables patients to recover quickly after the operation. Sepsis after PCNL is generally urinary sepsis, with an incidence rate of 1.4% to 4.4%. If not diagnosed and treated in time, it can easily cause septic shock and increase the risk of death for patients [1][2]. Early diagnosis of postoperative sepsis in patients with PCNL is conducive to the implementation of subsequent targeted treatment measures and can reduce the mortality rate. Relevant reports indicate that, in the initial stage of sepsis, one of the main features is the extensive expression of inflammatory mediators and immune activation. As the disease progresses, the body may experience a gradual decline in immune function [3]. The soluble trigger receptor-1 (sTREM-1) of myeloid cells, an immune-related marker, is closely associated with the severity of infection [4]. It has been reported that the immune indicator neutrophil-to-lymphocyte ratio (NLR) can be used to predict postoperative sepsis in patients with PCNL [5].

Percutaneous nephrolithotomy (PCNL) is a commonly used minimally invasive surgery for the treatment of kidney stones. Still, postoperative sepsis is one of its serious complications, which may lead to a high mortality rate and complex clinical management. In recent years, the potential value of immune indicators and cytokines in diagnosing sepsis has garnered widespread attention. These biomarkers can help doctors identify and manage sepsis patients earlier. Previous studies have shown that specific immune indicators, such as C-reactive protein (CRP), procalcitonin (PCT), and various cytokines, are involved in this process. These indicators are closely related to the severity and prognosis of sepsis. Domestic research also supports these findings and further explores the application value of other novel markers, such as myeloperoxidase (MPO) and soluble CD14 subtype (sCD14-ST), in the diagnosis of sepsis [6].

Although numerous studies have explored the role of immune indicators and cytokines in sepsis, their specific manifestations and diagnostic value in patients with sepsis after PCNL still need further verification. Therefore, we aimed to evaluate the diagnostic value of these immune indicators and cytokines in sepsis patients after PCNL. A systematic analysis of their changing characteristics provides clinicians with more reliable diagnostic tools and a basis for treatment, thereby improving patient prognosis.

## Materials and methods

### General information

Clinical information was gathered from 405 patients with calculi who underwent PCNL at our facility between January 2021 and December 2024. Based on whether sepsis developed following the procedure, the patients were divided into two groups: 12 patients developed sepsis, and 393 patients did not. The diagnostic criteria for sepsis were as follows: a positive blood bacterial culture, clinically confirmed urinary sepsis, and clinical symptoms related to a urinary tract infection with a score of less than 15 points (meeting any two of these criteria).

The inclusion criteria were as follows: ① aged 18 years; ② received unilateral PCNL treatment; and ③ had complete medical records.

The exclusion criteria were as follows: ① patients with a history of PCNL, urethral stent placement, or nephrostomy tube placement; ② patients with concurrent mental disorders; ③ patients with cancer; ④ patients with severe organ (heart, liver, lung, etc.) diseases, coagulation disorders, autoimmune diseases, etc.; and ⑤ Patients whose sepsis occurred before the operation or antibiotic treatment was received due to high fever.

### Index detection

On the second day after the operation, peripheral blood was drawn from the patients, and the NLR was determined via an automatic blood cell analyser (Mission HA-360) from Aikang Biotechnology. CD4+ and CD8+ cells were detected via flow cytometry, and the CD4+/CD8+ ratio was calculated. An enzyme-linked immunosorbent assay was used to measure the levels of serum sTREM-1, PCT, inter-leukin-6 (IL-6), and tumour necrosis factor-α (TNF-α) via an automatic biochemical analyser (Hitachi 7600).

### Statistical methods

The data were processed via SPSS 26.0 statistical software. Count data are expressed as the number of cases, and the χ^2^ test was used. The independent sample t-test was employed for group comparisons, and measurement data that followed a normal distribution are reported as (x̄ ± s). Receiver operating characteristic (ROC) curves were used to evaluate the ability of each index to predict sepsis. P values less than 0.05 were regarded as statistically significant.

## Results

### Comparison of the general characteristics of the two patient groups

Age, sex, body mass index (BMI), smoking and drinking history, stone location, diabetes status, hypertension status, and the long diameter of stones did not differ significantly (all P > 0.05). The number of patients with staghorn calculi in the sepsis group was considerably higher than that in the nonsepsis group (P < 0.05), as shown in Table 1.

**Table 1:** Comparison of general data of the two groups of patients with calculi.

Group	Cases	Gender		Age<br>(years)	BMI<br>(kg/m^2^)	Smoking	History<br>of alcohol<br>consumption	Location of the<br>stone (Example)		Hypertension	Diabetes	Antler-shaped<br>stones	Long<br>diameter<br>of calculi	
		Male	Female					Renal	Ureter				≤2.5cm	>2.5cm
Without<br>the<br>sepsis<br>group	393	201	192	50.98±8.23	23.27±2.38	54	64	357	36	110	54	56	44	349
Sepsis<br>group	12	7	5	52.36±8.74	23.45±2.36	3	2	10	2	3	2	7	2	10
x^2^/t<br>value	-	0.241		80.571	0.258	0.467	0.131	0.141		0.010	0.018	14.035	0.016	
P value	-	0.624		0.568	0.796	0.494	0.718	0.707		0.921	0.892	<0.001	0.899	

### Comparison of immune indicators and cytokine levels between the two groups of patients

The NLR and sTREM-1 levels were greater in the sepsis group than in the nonsepsis group (P<0.05), and the CD4+/CD8+ ratio was significantly lower in the sepsis group than in the nonsepsis group (P<0.05), see Table 2. The levels of serum IL-6, TNF-α and PCT in the sepsis group were significantly greater than those in the nonsepsis group (all P<0.05, Table 3).

**Table 2:** Comparing the Two Groups of Stone Patients' Immune Indicators (x̄ ± s).

Group	Number of cases	CD4+/CD8+	NLR	STREM-1 (μg/L)
Without the sepsis group	393	1.01±0.18	1.96±0.31	0.82±0.14
Sepsis group	12	0.78±0.12	2.58±0.52	1.05±0.17
t value		4.394	6.662	5.570
P value		<0.001	<0.001	<0.001

**Table 3:** Comparison of Cytokines between the Two Groups of Stone Patients (x̄ ± s).

Group	Number of cases	IL6 (ng/L)	TNF-α (ng/L)	PCT (μg/L)
Without the sepsis group	393	11.48±1.87	19.32±3.74	1.45±0.27
Sepsis group	12	14.75±2.59	23.46±4.13	1.89±0.31
t value	-	5.894	3.766	5.537
P value	-	<0.001	<0.001	<0.001

### The predictive value of the CD4+/CD8+ ratio, NLR, sTREM-1, IL-6, TNF-α and PCT for sepsis after PCNL

Compared with the individual detection of CD4+/CD8+ T cells and the NLR, sTREM-1, IL-6, TNF-α, and PCT, the AUC of the combined prediction of sepsis after PCNL was 0.996, with a predictive sensitivity of 91.3% and a specificity of 98.4%, see Table 4.

**Table 4:** ROC curve analysis for predicting sepsis after PCNL.

Variable	AUC	Standard<br>error	P value	95% CI		Optimal cutoff<br>value	Sensitivity (%)	Specificity (%)
				Upper limit	Lower limit			
CD4+/CD8	0.757	0.046	0.002	0.666	0.847	0.965	91.7	60.2
NLR	0.754	0.069	0.003	0.618	0.890	2.265	61.6	84.5
sTREM-1	0.918	0.033	<0.001	0.853	0.983	0.945	91.5	81.9
IL-6	0.857	0.041	<0.001	0.776	0.938	13.255	91.7	66.9
TNF-a	0.711	0.071	0.013	0.571	0.850	22.395	66.7	73.8
PCT	0.849	0.057	<0.001	0.738	0.960	1.502	91.4	74.0
United	0.996	0.003	<0.001	0.000	1.000	-	91.3	98.4

## Discussion

During PCNL surgery, the crushing of stones can release bacteria and endotoxins, which flow back into the bloodstream through lymphatic vessels and the corresponding filtration system, causing organ dysfunction and leading to sepsis [7][8]. Because most antler stones are infectious and usually contain many urease bacteria, they are more likely to become sources of infection. On the other hand, staghorn stones may damage the mucopolysaccharide protective layer in the urinary tract mucosal tissue, increasing the incidence of sepsis.

Currently, in clinical practice, sepsis is believed to be caused by either retrograde or hematogenous infection with bacteria and toxic substances. Within a relatively short period of time, nonspecific antibacterial substances (including immunoglobulins, white blood cells, and macrophages) are consumed in large quantities, and many inflammatory mediators are released throughout the body, resulting in damage to vascular endothelial cells and causing microcirculation disorders and even organ dysfunction [9][10].

Studies have shown that patients with sepsis have certain immune function abnormalities, and precise immunotherapy is currently a research hotspot in the field of immunotherapy for patients with sepsis [11]. In the classification of T lymphocytes, CD4+ cells are known as T helper cells and play a central role in the immune response system. CD8+ T cells are T suppressor cells and T killer cells and are effector cells of the immune response system. An increase in CD8+ cells, a decrease in CD4+ cells, and an imbalance between CD4+ and CD8+ cells indicate that the body's immune system has been compromised [12]. In this study, the CD4+/CD8+ T-cell ratio in the sepsis group was significantly lower compared to that in the nonsepsis group. The CD4+/CD8+ ratio exhibited a relatively high predictive sensitivity for sepsis, reaching 91.7%, which was attributed to persistent changes in immune function that weakened in early sepsis. Studies have shown that sTREM-1 can assist in the early diagnosis of sepsis in elderly burn patients [13]. Some studies have noted that the NLR plays a particular role in predicting the condition and prognosis of patients with sepsis [14]. The immune function of patients with sepsis after PCNL is disordered to a certain extent, resulting in significant changes in the counts of neutrophils and lymphocytes, which aggravate the infection and ultimately worsen the condition. A considerable increase in neutrophils can damage the parenchymal cells of organs, causing organ dysfunction. In severe cases, it can lead to multiple organ dysfunction. TREM-1 is a member of the immunoglobulin family. sTREM-1 is its dissolved form. When sepsis occurs, phagocytes are activated to synthesise in large quantities and enter body fluids, leading to an increase in the level of sTREM-1. IL-6 is synthesised mainly in mononuclear macrophages, T cells, glial cells, fibroblasts and endothelial cells. Monocytes, macrophages and T cells secrete TNF-α. Both IL-6 and TNF-α are proinflammatory cytokines [15][16]. The expression level of PCT is closely related to the inflammatory response caused by microbial infection - the more severe the inflammation, the higher the level [17]. The mechanism of elevated IL-6 levels may be as follows: (1) Patients with sepsis have cellular immune deficiency, and the regulation of the ratio of T cells to B cells is disrupted, thereby increasing the number of circulating immune complexes in the serum. (2) During the process of macrophages phagocytosing corresponding antigenic substances, IL-6 and TNF-α act only on vascular endothelial cells. Under pathological conditions, IL-6 and TNF-α are key inflammatory factors in the pathogenesis, causing pathological damage and accelerating the occurrence and progression of disease [18][19][20]. Elevated levels of TNF-α can intensify the body's inflammatory response, increase vascular permeability, cause cellular dysfunction, microcirculation disorders, and hemodynamic abnormalities, ultimately leading to organ dysfunction [21][22][23][24]. The level of PCT in the serum of normal people is extremely low. When systemic bacterial infection occurs, it can significantly increase within 4 hours and reach its peak at 6 hours. It is relatively sensitive to sepsis [25][26][27][28].

Through ROC curve analysis in this study, it was found that the combined aUc of CD4+/CD8+, NLR, sTREM-1, IL-6, TNF-α, and PCT for predicting sepsis was 0.996, indicating a relatively high predictive efficacy. The sensitivity and specificity were 91.3% and 98.4%, respectively, indicating that this method could be used for predicting sepsis after PCNL. In conclusion, the occurrence of sepsis after PCNL can significantly increase the NLR, sTREM-1, IL-6, TNF-α, and PCT levels, while also significantly decreasing the CD4+/CD8+ ratio. Their combined detection can assist in the early diagnosis of sepsis and has a high detection value.

## Dodatak

### Authors' contribution

Yiheng Jin and Yamei Li made equal contributions to the work.

### Acknowledgments

The authors would like to express their gratitude to AJE and Yuesheng Press Language Editing Services for the expert linguistic services provided.

### Conflict of interest statement

All the authors declare that they have no conflict of interest in this work.
